# Warming experiments elucidate the drivers of observed directional changes in tundra vegetation

**DOI:** 10.1002/ece3.1499

**Published:** 2015-04-12

**Authors:** Robert D Hollister, Jeremy L May, Kelseyann S Kremers, Craig E Tweedie, Steven F Oberbauer, Jennifer A Liebig, Timothy F Botting, Robert T Barrett, Jessica L Gregory

**Affiliations:** 1 Biology Department, Grand Valley State University1 Campus Drive, Allendale, Michigan, 49401; 2 Department of Biological Sciences, Florida International University11200 SW 8th Street, Miami, Florida, 33199; 3 Department of Biological Sciences, University of Notre DameNotre Dame, Indiana, 46556; 4 Department of Biology, University of Texas at El PasoEl Paso, Texas, 79968

**Keywords:** Arctic, biodiversity, *Cassiope tetragona*, climate change, community change, ITEX, *Poa arctica*

## Abstract

Few studies have clearly linked long-term monitoring with in situ experiments to clarify potential drivers of observed change at a given site. This is especially necessary when findings from a site are applied to a much broader geographic area. Here, we document vegetation change at Barrow and Atqasuk, Alaska, occurring naturally and due to experimental warming over nearly two decades. An examination of plant cover, canopy height, and community indices showed more significant differences between years than due to experimental warming. However, changes with warming were more consistent than changes between years and were cumulative in many cases. Most cases of directional change observed in the control plots over time corresponded with a directional change in response to experimental warming. These included increases in canopy height and decreases in lichen cover. Experimental warming resulted in additional increases in evergreen shrub cover and decreases in diversity and bryophyte cover. This study suggests that the directional changes occurring at the sites are primarily due to warming and indicates that further changes are likely in the next two decades if the regional warming trend continues. These findings provide an example of the utility of coupling in situ experiments with long-term monitoring to accurately document vegetation change in response to global change and to identify the underlying mechanisms driving observed changes.

## Introduction

Identifying the drivers of documented change in natural ecosystems is a challenge due to the many changing abiotic and biotic factors occurring at a given location and over time (Jeltsch et al. 2008; Thuiller et al. 2008). Yet if reasonable forecasts are to be made, it is critical that the primary driver(s) be identified. Determining clearly whether climate change is the primary driver is especially challenging due to the large variability in weather between years. Arctic ecosystems have been studied intensively to determine the impacts of climate change because warming in the Arctic has been documented since the 1800s and has been occurring at faster rates in recent decades (Kaufman et al. 2009; IPCC 2013). The response of arctic plant communities to climate change is of particular interest for the following reasons. Small changes in environmental conditions can have large effects on the plant community (Billings 1952; Chapin et al. 1995). These changes in plant community dynamics have been associated with alterations in ecosystem function and nutrient cycling (Shaver and Chapin 1991; Hobbie and Chapin 1998). Alterations to plant community structure can have far-reaching consequences as they provide shelter for animals and are the base of the food web (Sørensen et al. 2008; Joly et al. 2010; Tape et al. 2010). Changes in canopy structure and physiology of plants could greatly influence the energy balance, which can impact regional climate and permafrost dynamics (Chapin et al. 2005). Finally, shifts in community dynamics and changes in ecosystem function have the potential to shift Arctic tundra ecosystems from a carbon sink to a source (Oechel et al. 1993) that could provide a significant feedback to climate change.

Many studies have been conducted to examine how tundra plant communities respond to environmental changes, such as increased temperatures and nutrient availability (Arft et al. 1999; Dormann and Woodin 2002; Callaghan et al. 2004). Yet most studies span 5 years or less and are unable to address whether or not plant community responses are maintained in the long term. Few studies address the tundra vegetation changes that occur after prolonged periods of environmental change (Hudson et al. 2011; Elmendorf et al. 2012a; Michelsen et al. 2012). Earlier studies have given insights into how plant communities may shift beyond the initial responses to changes in their environment (Chapin et al. 1995; Hollister et al. 2005). Temperature gradient studies, paleoecological investigations, and modeling efforts clearly show that with warming, tundra vegetation moves from an open canopy (with limited vascular plant cover) toward a closed canopy that gets taller due to the increased abundance of graminoids, then shrubs, and, ultimately with enough warming, trees (Oechel et al. 1997). Given that preexisting plant communities can resist change (Hudson and Henry 2010; Svenning and Sandel 2013), the ultimate question is as follows: *Will modest warming cause clear directional change in tundra communities, and if so, how quickly will the change occur?*

To answer this question, researchers have conducted warming experiments throughout many regions of the world (Rustad et al. 2001). The tundra has received special attention for the reasons listed above and most of the warming studies in tundra collaborate as part of the International Tundra Experiment (ITEX) network (http://ibis.geog.ubc.ca/itex/). ITEX researchers have agreed on standard protocols that allow for detailed comparisons across sites. The primary focus has been on documenting the response of tundra vegetation to passive experimental warming imposed by open-top chambers. Now that many of the sites have monitored vegetation for over a decade, changes in the control plots have become increasingly important for documenting the impact of climate change (Elmendorf et al. 2012b). The goal of this study is to document the effects of experimental warming on plant community dynamics over nearly two decades and to evaluate whether the changes in plant communities associated with this experimental warming are consistent with the natural trends observed in the control plots. Specifically, we examined changes at the species level in plant cover, canopy height, and species diversity at four sites which span a moisture and climate gradient. We focus on the consistency of the response over time to look for clear directional trends that the community may be moving toward.

## Materials and Methods

### Site descriptions

**Figure 1:**
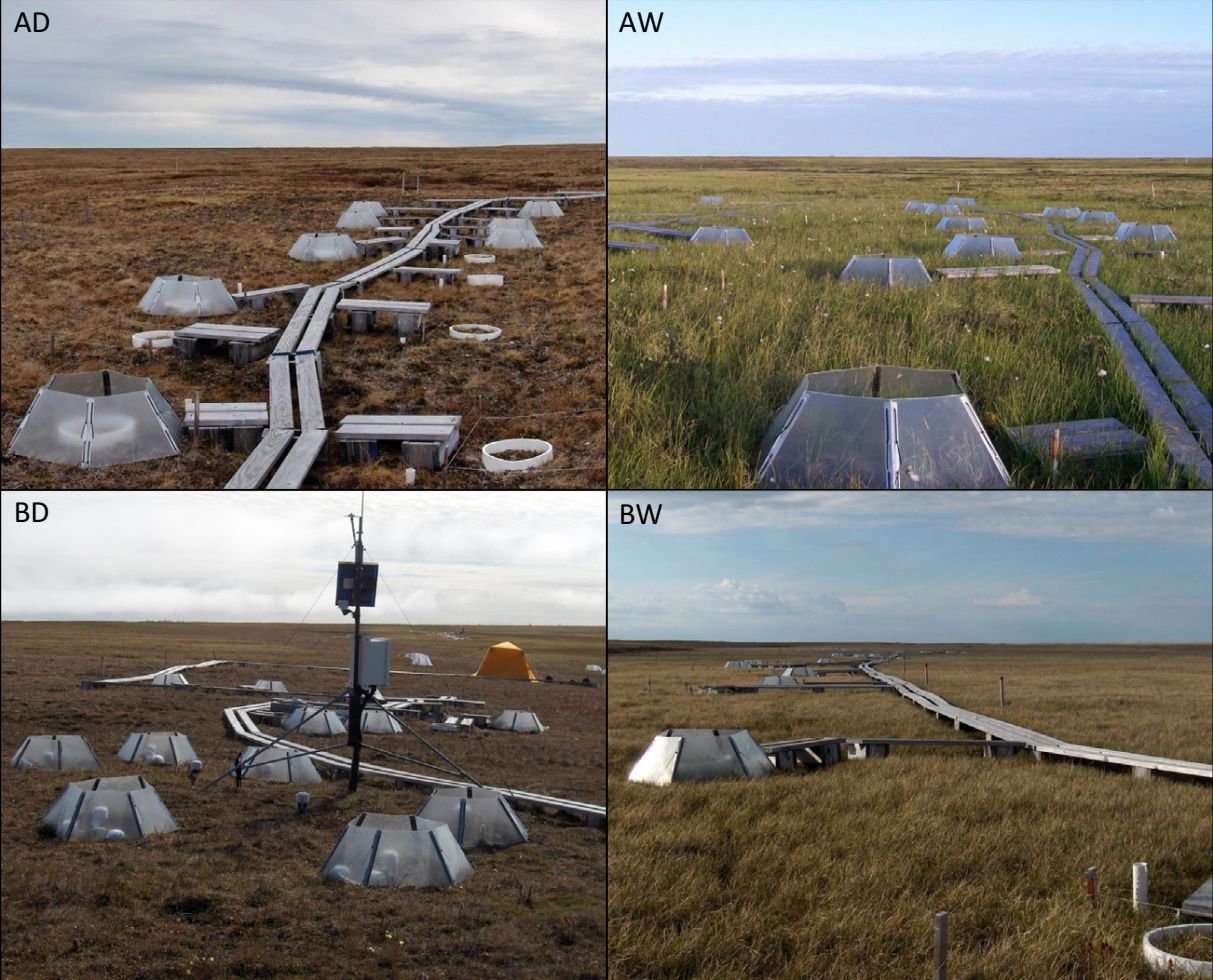
Images of the four sites: Atqasuk dry (AD), Atqasuk wet (AW), Barrow dry (BD), and Barrow wet (BW).

This study consisted of four study sites; two sites were located near Barrow, AK (71°19′N, 156°36′W), and two were approximately 100 km south near Atqasuk, AK (70°27′N, 157°24′W) (Fig.1). At each location, a wet and a dry site were established. These locations are representative of two bioclimate zones; Barrow is classified in the circumpolar vegetation map (CAVM 2003) and by Raynolds et al. (2006) as Biozone C and Atqasuk as Biozone D. Both locations have a deep heritage of research; Barrow was an International Biological Tundra Biome site in the early 1970s (Brown et al. 1980), and Atqasuk was the focus of the Research on Arctic Tundra Environments (Batzli 1980). The sites near Barrow include a dry (BD) and wet (BW) site; both have a mean July temperature of ∼4°C (Brown et al. 1980). In Barrow, snowmelt occurs in early to mid-June and maximum thaw depth is typically between 50 and 100 cm. The BD site is situated on a well-drained beach ridge above a drained thaw lake dominated by *Cassiope tetragona*, *Salix rotundifolia*, and *Luzula confusa*. The BW site is in a frequently inundated transitional zone between the beach ridge of the dry site and a drained lake basin, and is dominated by *Carex aquatilis*, *Dupontia fisheri*, and *Eriophorum spp*. The sites near Atqasuk also include a dry (AD) and wet (AW) site; both have a mean July temperature of ∼9°C (Batzli 1980). Snowmelt in Atqasuk occurs in late May, and maximum thaw depth is typically between 90 and 120 cm. The AD site is on a well-drained ridge above a thaw lake and is dominated by *Cassiope tetragona*, *Ledum palustre*, and *Luzula confusa*. The AW site is located at the edge of a thaw lake in a frequently inundated meadow and is dominated by *Carex aquatilis*, *Eriophorum spp*., and *Salix pulchra*. Topographic changes are small (<0.5 m) at the sites; however, even small differences may be associated with significant shifts in plant community composition and soil moisture (Webber 1978; Komárková and Webber 1980).

The four sites were established between 1994 and 1996 and have been monitored since using standard ITEX protocols. Each site consists of 48 ∼1 m^2^ plots (24 control and 24 warmed). Warming was achieved using hexagonal open-top chambers (OTCs) constructed of Sun-Lite HPTM fiberglass according to the guidelines in the ITEX manual (Molau and Mølgaard 1996). OTCs were installed every year shortly after snowmelt and removed at the end of the growing season. OTCs have been shown to warm the surface air temperature an average of 0.6 to 2.2°C (Hollister et al. 2006), and, despite experimental artifacts (Bokhorst et al. 2013), they have been shown to realistically simulate climate change in the tundra (Hollister and Webber 2000).

### Climate monitoring

Weather stations were established in 1998 at the dry sites at both Barrow and Atqasuk. Readings of temperature at screen height (2 m, 107 temperature probe) and precipitation (35 cm, TE525 tipping bucket rain gage) were taken every 15 min, averaged (temperature) or summed (precipitation), and recorded every hour (CR10X datalogger; the above instruments were produced by Campbell Scientific Inc., Logan, UT). At each of the four sites, two plots were also monitored for soil moisture at 7.5 cm depth (HYD-10-A hydra probe, Stevens Vitel Hydrological and Meteorological Systems, Chantilly, VA). Voltages from the soil moisture probe were recorded every hour and were converted to water fraction by volume (WFV). The focus of the measurements was relative change between years; thus, readings were not calibrated with gravimetric methods. During times prior to the weather station establishment or instrument malfunction, readings from a nearby station were substituted (for details see Hollister et al. 2006).

### Vegetation sampling

All four sites were sampled four separate times (1995–97, 2000, 2007–08, and 2012; Table1) according to the nondestructive point frame method outlined in the ITEX Manual (Molau and Mølgaard 1996). A 75 cm^2^ 100-point grid with measurement points every 7 cm was leveled above the plant canopy using permanent markers that allow for reasonably accurate resampling of the same point over multiple years. At each point on the grid, a graduated ruler was lowered to the first contact (uppermost) within the plant canopy and then to the lowermost contact at that point. This shortcut, omitting intermediate contacts, has been shown to be effective at detecting vegetation change in tundra communities, especially at sites with a leaf area index less than two; however, it does artificially limit cover to 200% (May and Hollister 2012). At each contact, the species, live/dead status, and height were recorded. Some species were difficult to identify in situ and were grouped into the closest secure taxon; this included only recording cryptograms to growth form (i.e., acrocarpous moss). Taxa were also grouped into broad growth forms (i.e., bryophytes) for analysis of growth form trends (see Table2 for grouping schemes).

**Table 1:** The years when vegetation was sampled and the associated number of summers of warming between samplings (cumulative number of years of warming in parentheses). Sites are Atqasuk dry (AD), Atqasuk wet (AW), Barrow dry (BD), and Barrow wet (BW)

Site	Year				Summers of warming			
	Sampling 1	Sampling 2	Sampling 3	Sampling 4	Sampling 1	Sampling 2	Sampling 3	Sampling 4
AD	1997	2000	2007	2012	2 (2)	3 (5)	7 (12)	5 (17)
AW	1997	2000	2007	2012	2 (2)	3 (5)	7 (12)	5 (17)
BD	1995	2000	2008	2012	2 (2)	5 (7)	8 (15)	4 (19)
BW	1996	2000	2008	2012	2 (2)	4 (6)	8 (14)	4 (18)

**Table 2:** Change in plant cover over time in control plots and in response to warming at the four sites. The average cover and standard error are presented at sampling 1, sampling 2, sampling 3, and sampling 4 for control (C1, C2, C3, and C4) and experimentally warmed (E1, E2, E3, and E4) plots. For convenience, the warming response is also presented as the differences between control and experimental plots at the four samplings (W1, W2, W3, and W4). The change over time in response to the ambient environment and to experimental warming is categorized as no change (.), inconsistent change (I), nondirectional change (N), and cumulative directional change (D^+^ – increase, D^−^ – decrease); because the response to warming could also be considered relative to the change in the control plots, the warming response could be categorized as consistent change (C^+^ – increase, C^−^ – decrease) and a cumulative directional change observed only in relation to the control plots is noted with an italicized *D* (see methods for further details). Taxa include all growth forms (in bold) present at a site and vascular plant species or narrower growth from (for nonvascular plants) that occurred in at least half the plots

Taxa	C1	C2	C3	C4		E1	E2	E3	E4		W1	W2	W3	W4
Atqasuk dry (AD) site														
**Deciduous Shrub**	**0.5 (0.3)**	**0.4 (0.3)**	**0.6 (0.4)**	**0.4 (0.2)**	**.**	**0.3 (0.2)**	**0.3 (0.2)**	**0.5 (0.2)**	**0.6 (0.4)**	**.**	**−0.3**	**−0.1**	**−0.2**	**0.2**
**Evergreen Shrub**	**29.2 (1.5)**	**22.6 (1.5)**	**35.5 (1.7)**	**23.2 (2.1)**	**N**	**29.8 (2.3)**	**26.1 (2.4)**	**33.0 (1.7)**	**26.9 (1.9)**	**.**	**0.6**	**3.5**	−**2.5**	**3.8**
*Cassiope tetragona*	6.3 (0.5)	4.7 (0.5)	6.0 (0.6)	3.3 (0.7)	N	7.2 (0.9)	5.5 (0.8)	7.7 (1.2)	6.2 (0.9)	*D*^+^	0.9	0.8	1.7	2.8
*Diapensia lapponica*	3.8 (0.7)	2.2 (0.7)	3.8 (0.7)	2.6 (0.6)	.	3.5 (0.6)	3.3 (0.5)	3.9 (0.6)	2.5 (0.5)	.	−0.2	1.0	0.1	−0.1
*Ledum palustre*	11.5 (1.6)	9.8 (1.6)	14.5 (1.6)	9.8 (1.3)	.	11.9 (1.6)	10.6 (1.8)	13.8 (1.6)	11.5 (1.3)	.	0.3	0.8	−0.7	1.7
*Vaccinium vitis-idaea*	7.6 (0.8)	6.0 (0.8)	11.3 (1.4)	7.4 (1.1)	N	7.2 (0.8)	6.8 (0.7)	7.6 (0.7)	6.7 (0.9)	.	−0.4	0.8	−3.6	−0.7
**Forb**	**0.7 (0.3)**	**0.4 (0.3)**	**0.8 (0.3)**	**0.6 (0.2)**	**.**	**0.7 (0.2)**	**0.8 (0.3)**	**1.5 (0.4)**	**1.4 (0.5)**	**.**	**0.0**	**0.5**	**0.7**	**0.8**
**Graminoid**	**12.3 (1.7)**	**9.0 (1.7)**	**18.3 (1.9)**	**10.7 (1.4)**	**N**	**12.6 (1.6)**	**6.4 (1.0)**	**15.9 (1.4)**	**9.2 (1.4)**	**.**	**0.4**	−**2.6**	−**2.5**	−**1.5**
*Hierochloe alpina*	3.0 (0.8)	1.4 (0.8)	3.3 (0.8)	2.3 (0.5)	.	2.8 (0.7)	1.3 (0.3)	4.9 (0.9)	2.2 (0.5)	.	−0.1	−0.1	1.6	−0.1
*Luzula confusa*	5.3 (0.8)	4.4 (0.8)	9.4 (1.1)	3.6 (0.6)	N	6.3 (1.1)	3.1 (0.6)	7.1 (1.1)	3.4 (0.7)	.	0.9	−1.3	−2.3	−0.3
**Bryophyte**	**9.8 (0.8)**	**12.0 (0.8)**	**7.9 (0.7)**	**6.4 (0.6)**	**N**	**10.5 (1.3)**	**11.3 (1.1)**	**7.9 (1.0)**	**6.5 (1.0)**	**.**	**0.7**	−**0.7**	**0.0**	**0.1**
Acrocarpous Moss	8.0 (0.7)	10.2 (0.7)	4.5 (0.6)	5.3 (0.6)	N	7.2 (0.9)	8.3 (0.9)	4.6 (0.8)	5.3 (0.9)	.	−0.8	−2.0	0.2	0.0
**Lichen**	**60.1 (1.8)**	**56.3 (1.8)**	**39.3 (2.3)**	**23.8 (1.7)**	**D**^−^	**67.2 (2.2)**	**55.8 (3.5)**	**37.4 (2.3)**	**18.9 (1.6)**	**D**^−^	**7.0**	−**0.5**	−**2.0**	−**4.9**
Crustose Lichen	1.8 (0.3)	8.1 (0.3)	2.1 (0.4)	4.8 (0.8)	N	1.5 (0.3)	7.8 (1.0)	2.3 (0.5)	4.3 (0.8)	.	−0.3	−0.3	0.1	−0.6
Foliose Lichen	16.6 (1.2)	12.3 (1.2)	10.7 (0.8)	5.8 (0.7)	D^−^	15.9 (1.1)	8.5 (0.8)	9.2 (0.9)	4.8 (0.6)	C^−^	−0.8	−3.8	−1.5	−1.1
Fruticose Lichen	41.8 (1.7)	36.0 (1.7)	26.5 (2.1)	13.2 (1.1)	D^−^	49.8 (1.9)	39.5 (2.6)	25.9 (1.8)	9.9 (1.0)	D^−^	8.0	3.5	−0.6	−3.3
Atqasuk wet (AW) site														
**Deciduous Shrub**	**8.0 (1.5)**	**8.6 (1.5)**	**8.0 (1.5)**	**7.8 (1.2)**	**.**	**6.2 (0.9)**	**8.0 (1.2)**	**7.0 (0.9)**	**7.5 (0.9)**	**.**	−**1.8**	−**0.6**	−**1.0**	−**0.3**
*Salix pulchra*	6.5 (1.5)	7.3 (1.5)	5.5 (1.1)	5.0 (1.1)	.	5.3 (0.9)	6.6 (1.2)	6.0 (0.8)	4.7 (1.0)	.	−1.3	−0.7	0.5	−0.3
**Forb**	**0.5 (0.2)**	**0.5 (0.2)**	**0.3 (0.1)**	**0.1 (0.1)**	**.**	**0.5 (0.2)**	**0.3 (0.2)**	**0.2 (0.1)**	**0.2 (0.1)**	**.**	**0.0**	−**0.1**	−**0.2**	**0.1**
**Graminoid**	**27.8 (1.5)**	**19.7 (1.5)**	**32.8 (1.4)**	**26.8 (1.1)**	**N**	**26.5 (1.5)**	**23.6 (1.8)**	**40.0 (1.7)**	**32.3 (1.8)**	**I**	−**1.3**	**3.9**	**7.1**	**5.5**
*Carex aquatilis*	19.8 (1.2)	12.6 (1.2)	24.5 (1.2)	18.1 (1.1)	N	18.5 (1.2)	15.2 (1.0)	30.1 (1.1)	23.0 (1.4)	I	−1.3	2.6	5.6	4.9
*Eriophorum angustifolium*	3.3 (0.8)	4.5 (0.8)	4.6 (0.8)	4.9 (0.6)	.	3.1 (0.6)	3.6 (0.7)	4.9 (0.7)	5.7 (0.7)	.	−0.2	−0.9	0.3	0.8
*Eriophorum russeolum*	4.5 (0.8)	2.4 (0.8)	2.8 (0.5)	3.2 (0.4)	.	4.5 (0.6)	4.5 (0.8)	3.8 (0.6)	3.1 (0.6)	.	0.1	2.1	1.0	−0.1
**Bryophyte**	**87.8 (0.9)**	**91.8 (0.9)**	**86.5 (2.6)**	**55.0 (3.1)**	**N**	**86.7 (1.2)**	**90.8 (1.4)**	**94.1 (0.8)**	**47.9 (3.0)**	**I**	−**1.1**	−**1.1**	**7.6**	−**7.1**
Acrocarpous Moss	31.5 (4.1)	32.0 (4.1)	29.5 (3.9)	22.5 (3.4)	.	31.8 (3.4)	31.0 (4.6)	29.0 (3.4)	17.0 (2.5)	.	0.2	−1.0	−0.5	−5.4
Pleurocarpous Moss[Table-fn tf2-1]	49.7 (3.9)	54.9 (5.2)	48.8 (5.5)	27.1 (2.7)	N	48.6 (3.1)	55.9 (4.2)	56.9 (3.6)	24.0 (2.6)	.	−1.1	1.0	8.1	−3.1
Sphagnum Moss	3.5 (0.9)	4.5 (0.9)	8.1 (1.8)	5.4 (1.5)	.	3.8 (1.4)	3.5 (1.6)	8.2 (2.7)	6.7 (2.7)	.	0.3	−1.0	0.0	1.3
**Lichen**	**1.0 (0.3)**	**0.6 (0.3)**	**0.3 (0.1)**	**0.5 (0.2)**	**.**	**1.0 (0.3)**	**0.3 (0.2)**	**0.2 (0.1)**	**0.3 (0.1)**	**.**	**0.0**	−**0.3**	−**0.1**	−**0.3**
Barrow Dry (BD) site														
**Deciduous Shrub**	**15.0 (1.1)**	**28.5 (1.1)**	**24.5 (1.5)**	**17.1 (1.6)**	**N**	**14.9 (1.3)**	**24.3 (2.0)**	**20.0 (1.8)**	**14.7 (1.2)**	**C**^−^	−**0.1**	−**4.2**	−**4.5**	−**2.5**
*Salix rotundifolia*	15.0 (1.1)	28.5 (1.1)	24.5 (1.5)	17.1 (1.6)	N	14.9 (1.3)	24.3 (2.0)	20.0 (1.8)	14.7 (1.2)	C^−^	−0.1	−4.2	−4.5	−2.5
**Evergreen Shrub**	**11.4 (1.0)**	**20.4 (1.0)**	**16.7 (1.2)**	**26.8 (1.7)**	**N**	**15.2 (1.1)**	**24.8 (1.8)**	**23.1 (2.0)**	**33.0 (2.5)**	***D***^**+**^	**3.8**	**4.4**	**6.4**	**6.2**
*Cassiope tetragona*	11.3 (1.0)	19.8 (1.0)	16.6 (1.3)	26.5 (1.8)	N	15.2 (1.1)	24.8 (1.8)	23.1 (2.0)	33.0 (2.5)	*D*^+^	3.9	5.0	6.5	6.5
**Forb**	**4.5 (0.6)**	**7.7 (0.6)**	**7.2 (0.9)**	**5.5 (0.9)**	**N**	**4.3 (0.4)**	**6.6 (0.8)**	**10.9 (1.8)**	**7.0 (1.3)**	**.**	−**0.3**	−**1.0**	**3.8**	**1.5**
*Potentilla hyparctica*	2.0 (0.5)	2.4 (0.5)	3.0 (0.7)	2.5 (0.7)	.	1.8 (0.3)	1.2 (0.4)	4.0 (0.8)	3.0 (0.6)	.	−0.2	−1.3	1.1	0.4
**Graminoid**	**3.0 (0.6)**	**7.3 (0.6)**	**7.2 (0.7)**	**6.2 (0.8)**	**N**	**4.5 (0.8)**	**12.3 (1.9)**	**16.3 (1.8)**	**12.0 (1.4)**	**I**	**1.4**	**5.0**	**9.1**	**5.8**
*Luzula confusa*	1.3 (0.4)	3.0 (0.4)	3.6 (0.5)	2.3 (0.3)	N	1.3 (0.3)	2.8 (0.5)	4.4 (0.6)	2.2 (0.4)	.	0.0	−0.2	0.8	−0.1
*Poa arctica*	0.5 (0.1)	1.6 (0.1)	1.8 (0.3)	2.1 (0.4)	D^+^	0.6 (0.2)	3.8 (0.4)	6.2 (1.0)	5.8 (0.9)	D^+^	0.2	2.1	4.4	3.6
**Bryophyte**	**11.0 (1.0)**	**19.8 (1.0)**	**11.7 (1.0)**	**17.3 (1.9)**	**N**	**8.4 (0.9)**	**13.8 (1.4)**	**6.3 (0.9)**	**9.4 (1.4)**	**C**^−^	−**2.6**	−**6.0**	−**5.4**	−**7.9**
Acrocarpous Moss	7.5 (0.9)	11.8 (0.9)	7.7 (0.9)	7.9 (1.0)	N	5.4 (0.6)	9.6 (1.1)	3.6 (0.5)	5.6 (0.9)	C^−^	−2.1	−2.2	−4.1	−2.3
Pleurocarpous Moss	2.0 (0.6)	5.7 (0.6)	3.0 (0.7)	8.9 (1.8)	N	1.3 (0.5)	3.2 (0.8)	2.1 (0.5)	3.5 (1.0)	I	−0.7	−2.5	−0.9	−5.4
**Lichen**	**27.0 (1.2)**	**37.9 (1.2)**	**31.9 (2.0)**	**41.0 (1.9)**	**N**	**25.8 (2.0)**	**24.7 (2.7)**	**15.9 (1.9)**	**17.0 (2.3)**	**D**^−^	−**1.3**	−**13.2**	−**16.0**	−**24.1**
Foliose Lichen	6.3 (0.9)	8.5 (0.9)	8.5 (0.7)	9.0 (0.9)	.	6.0 (0.7)	6.0 (0.6)	4.3 (0.6)	5.2 (0.7)	*D*^−^	−0.3	−2.5	−4.3	−3.8
Fruticose Lichen	16.7 (1.0)	26.3 (1.0)	22.8 (1.6)	31.0 (1.7)	N	15.7 (1.6)	15.8 (2.2)	11.1 (1.6)	11.1 (1.9)	D^−^	−1.0	−10.5	−11.8	−19.9
Barrow Wet (BW) site														
**Deciduous Shrub**	**0.2 (0.1)**	**0.0 (0.1)**	**0.0 (0.0)**	**0.2 (0.1)**	**.**	**0.3 (0.2)**	**0.7 (0.4)**	**1.8 (1.0)**	**1.2 (0.6)**	**C**^**+**^	**0.2**	**0.6**	**1.8**	**1.0**
**Forb**	**17.8 (1.8)**	**14.6 (1.8)**	**13.2 (1.8)**	**8.5 (1.6)**	**D**^−^	**15.6 (1.7)**	**13.1 (1.9)**	**15.7 (2.0)**	**6.8 (0.9)**	**.**	−**2.2**	−**1.5**	**2.5**	−**1.8**
*Saxifraga cernua*	2.0 (0.4)	2.1 (0.4)	1.9 (0.5)	0.9 (0.2)	.	2.5 (0.4)	1.9 (0.4)	3.8 (0.7)	1.4 (0.3)	C^+^	0.4	−0.2	1.9	0.5
*Stellaria laeta*	4.0 (0.8)	2.1 (0.8)	1.8 (0.4)	1.3 (0.3)	N	4.0 (0.9)	3.1 (0.9)	1.6 (0.3)	1.1 (0.2)	.	0.0	1.0	−0.2	−0.2
**Graminoid**	**43.3 (1.8)**	**63.0 (1.8)**	**41.3 (2.4)**	**49.0 (2.2)**	**N**	**44.4 (1.2)**	**60.4 (6.8)**	**43.0 (2.2)**	**50.3 (2.8)**	**.**	**1.1**	−**2.5**	**1.7**	**1.3**
*Carex aquatilis*	18.5 (2.0)	14.6 (2.0)	19.0 (1.4)	20.3 (1.6)	N	23.1 (2.0)	16.6 (1.5)	26.6 (1.3)	25.5 (1.8)	C^+^	4.5	2.0	7.7	5.3
*Dupontia fisheri*	7.8 (0.9)	12.9 (0.9)	7.8 (1.0)	13.6 (2.0)	N	6.1 (0.8)	9.0 (1.4)	4.0 (0.6)	9.2 (1.5)	C^−^	−1.6	−3.8	−3.8	−4.4
*Eriophorum angustifolium*	9.9 (1.5)	19.0 (1.5)	4.9 (1.0)	7.8 (1.5)	N	8.4 (1.4)	18.6 (4.1)	4.0 (0.7)	7.0 (1.9)	.	−1.5	−0.4	−0.9	−0.8
*Eriophorum russeolum*	2.3 (0.4)	4.9 (0.4)	5.0 (0.6)	2.6 (0.6)	N	2.8 (0.6)	4.0 (0.9)	4.7 (0.7)	3.1 (0.7)	.	0.6	−0.9	−0.3	0.5
Poaceae spp.^2^	3.8 (0.7)	11.3 (0.7)	4.1 (0.8)	4.4 (0.7)	N	2.7 (0.4)	11.8 (1.5)	2.8 (0.7)	5.3 (0.9)	.	−1.1	0.5	−1.3	0.9
**Bryophyte**	**42.0 (3.1)**	**56.4 (3.1)**	**24.9 (3.0)**	**31.6 (4.2)**	**N**	**42.0 (4.1)**	**45.6 (3.3)**	**16.1 (2.2)**	**18.4 (3.0)**	**D**^−^	**0.0**	−**10.8**	−**8.8**	−**13.2**
Acrocarpous Moss	17.2 (1.9)	25.1 (1.9)	9.1 (1.3)	14.3 (2.4)	N	16.5 (2.7)	20.6 (2.7)	7.4 (1.0)	8.7 (1.8)	C^−^	−0.7	−4.5	−1.7	−5.6
Pleurocarpous Moss^1^	23.8 (2.2)	30.9 (2.9)	15.7 (2.7)	16.9 (4.0)	N	24.9 (3.0)	24.0 (2.1)	8.7 (1.7)	9.4 (2.0)	D^−^	1.1	−6.9	−7.0	−7.5
**Lichen**	**2.5 (0.8)**	**3.3 (0.8)**	**5.5 (1.8)**	**5.8 (2.1)**	**.**	**1.8 (0.7)**	**1.8 (0.9)**	**1.7 (0.7)**	**1.9 (0.8)**	***D***^−^	−**0.8**	−**1.5**	−**3.8**	−**3.9**
Foliose Lichen	2.5 (0.8)	3.3 (0.8)	5.5 (1.8)	5.8 (2.1)	.	1.6 (0.7)	1.7 (0.9)	1.6 (0.7)	1.8 (0.8)	*D*^−^	−0.9	−1.6	−3.8	−4.0

1 Pleurocarpous moss included leafy liverworts at the wet sites due to difficulties with identification underwater.

2 *Calamagrostis holmii, Hierochloe pauciflora, and Poa arctica* were lumped due to difficulties identifying sterile tillers.

### Data analysis

Cover, height, and diversity indices were calculated for each plot. All encounters of equipment (i.e., individual tags) were removed from the dataset before analysis (<1% total cover). Cover estimates were calculated by summing all contacts from each grouping examined (e.g., taxon, live contacts, dead contacts). The cover and canopy height of all taxa were based on live encounters only (except for litter, standing dead, and open canopy cover). Open canopy was calculated by summing the cover of all mosses, lichens, litter, and bare ground encountered in the top contacts only. Dead plant matter was considered standing dead if it was attached or litter if it was unattached. Height for each contact was calculated by taking the difference between the encountered plant contact and the ground contact. Canopy height was calculated using only the tallest encounter of each grouping (species, growth form or other) in each plot. Species richness and Shannon index were calculated per plot based on cover estimates of all live taxa using the computer program PC-ORD 4.0 (McCune and Mefford 1999).

**Figure 2:**
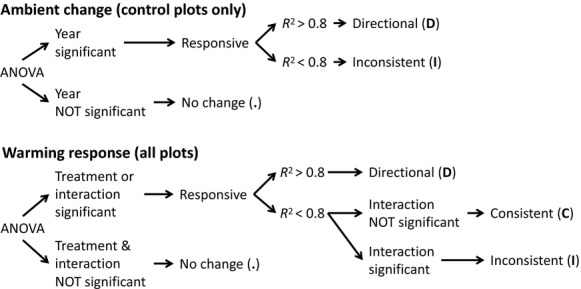
Decision tree used to determine the response categorization (see Methods for details).

The cover, height, and diversity indices at each sampling time were used to calculate estimates of vegetation change occurring in the control plots and due to warming. To determine whether the control plots were changing, a one factor repeated-measures ANOVA was performed (using year) on the control plots only (Fig.2). If the difference between years was significant, the taxon was considered “responsive” and a correlation was performed between year and the yearly average value to determine whether the change was directional. If the response was not directional, it was considered “nondirectional.” To determine whether the plants were responding to experimental warming, a 2 factor repeated-measures ANOVA was performed (using year, treatment, and the interaction between them; Fig.2). If treatment was significant or there was a significant interaction between year and treatment, then the taxon was considered “responsive.” To determine whether the response was directional, a correlation was performed between year and the yearly average value for experimental plots or between year and the yearly average difference between experimental and control plots. If the response was not considered directional, then it was considered “inconsistent” if there was a significant interaction between year and treatment or “consistent” if the interaction was not significant. All results were considered statistically significant with a Type 1 error probability of 5% or less using R version 2.13.1 statistical platform (R Development Core Team 2011). Repeated-measures ANOVAs were conducted using linear mixed effect models (the “lme” function in the R package “nlme”). Regressions between the average value of a year versus year (for significant responses) were considered “directional” if the *R*^2^ was greater than 0.8. Cases that varied significantly from normality were either log- or square-root-transformed or tested with an equivalent nonparametric test (Kruskal–Wallis).

## Results

### Temperature, precipitation, and soil moisture

**Figure 3:**
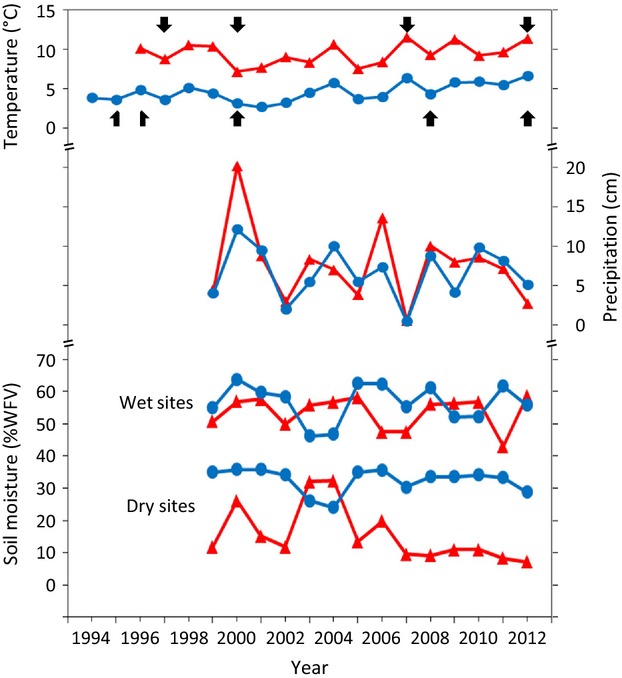
Mean temperature (top), total precipitation (middle), and mean soil moisture (bottom) at the sites in Atqasuk (red triangles) and Barrow (blue circles) in July during the years of the study. Years when vegetation was sampled are noted with arrows. No precipitation or soil moisture information was available before 1999.

Mean July temperature varied at both Atqasuk and Barrow throughout the duration of this study (Fig.3). Temperatures during the summers when the vegetation was sampled varied greatly. Both regions showed increasing temperature trends over the duration of the study, although neither trend was statistically significant. Precipitation and soil moisture also varied greatly between years (Fig.3), and the AD site has had consistent low soil moisture from 2007 through 2012.

### Change within sites

**Figure 4:**
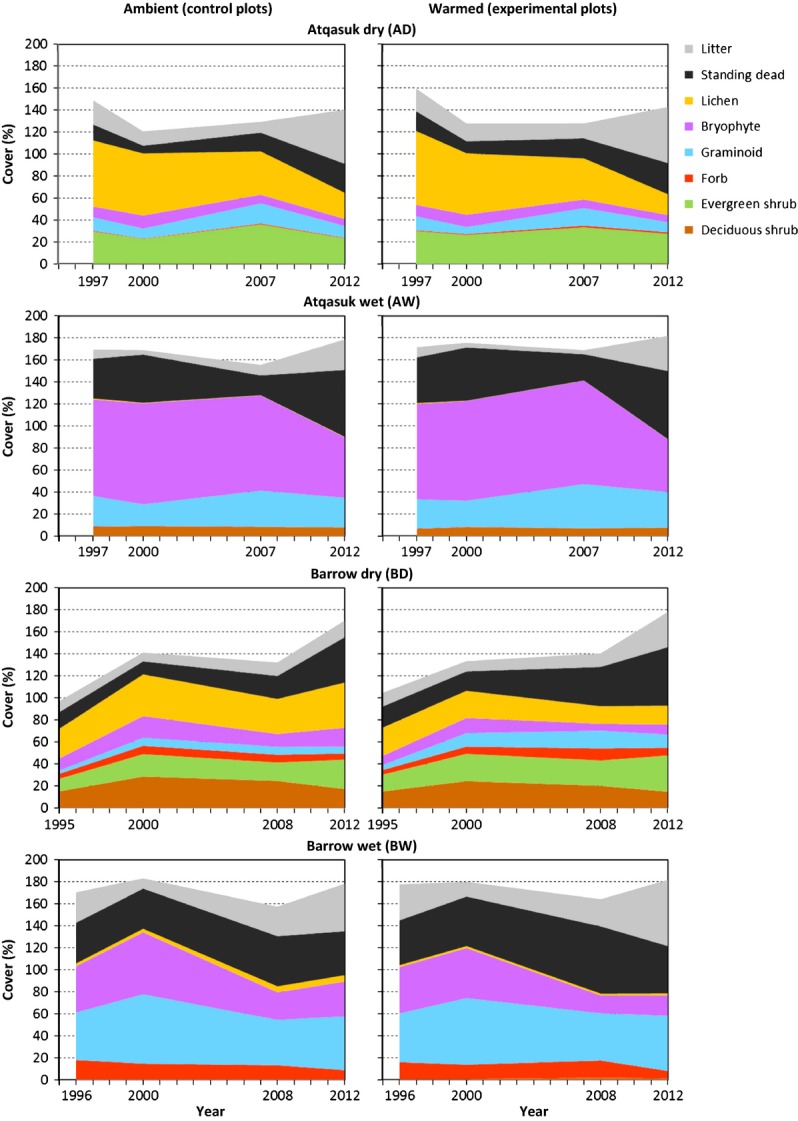
Changes in cover over time in the ambient environment and with warming at the four sites. Years sampled are shown on the axis.

At the AD site, lichens decreased over time in the control plots and with warming (Tables2 and 3, Fig.4). Vascular plant diversity decreased, whereas changes in the cover of evergreen shrubs, graminoids, bryophytes, total live plants, standing dead, litter, and open canopy and species richness were nondirectional over time (Tables2 and 3, Fig.4). Cover of *Cassiope tetragona* and standing dead increased with warming. Although not quantitatively measured, it was clear that the site was heavily impacted by caribou grazing the winter before the second sampling; the effect of this can be seen by the decrease in canopy heights and the decrease in the cover of total live plants and standing dead (Tables3 and 4).

**Table 3:** Change in community indices over time in control plots and in response to warming at the four sites (AD – Atqasuk dry, AW – Atqasuk wet, BD – Barrow dry, and BW – Barrow wet). The community indices used were cover of live, standing dead, and dead unattached (litter) plant material; the percent of the canopy that was not occupied by vascular plants (Open Canopy); and the diversity metrics species richness and Shannon index. See Table2 for an explanation of the table layout

Site	C1	C2	C3	C4		E1	E2	E3	E4		W1	W2	W3	W4
Total Live Plants														
AD	112.6 (2.3)	100.7 (2.3)	102.6 (3.1)	65.3 (2.7)	N	121.0 (3.2)	100.8 (4.6)	96.1 (2.3)	63.8 (2.6)	.	8.4	0.1	−6.5	−1.5
AW	125.0 (1.3)	121.2 (1.3)	128.0 (2.2)	90.5 (3.5)	N	121.0 (1.7)	122.9 (1.7)	141.4 (1.4)	88.4 (2.5)	I	−4.0	1.8	13.4	−2.1
BD	72.0 (1.0)	121.4 (1.0)	99.3 (1.8)	114.1 (2.4)	N	73.0 (1.5)	106.4 (3.9)	92.6 (1.8)	93.1 (2.7)	I	1.0	−15.0	−6.7	−21.0
BW	106.1 (3.2)	137.3 (3.2)	85.3 (2.2)	97.8 (5.0)	N	104.5 (4.7)	121.7 (6.8)	78.5 (2.9)	81.0 (3.7)	I	−1.6	−15.7	−6.8	−16.8
Standing Dead														
AD	14.6 (1.2)	7.1 (1.2)	17.1 (1.9)	26.3 (2.5)	N	18.0 (1.4)	11.0 (1.4)	18.2 (1.6)	28.3 (2.4)	C^+^	3.4	3.8	1.1	2.0
AW	36.2 (1.4)	43.8 (1.4)	18.0 (2.1)	60.8 (2.8)	N	41.5 (1.6)	48.5 (2.2)	24.0 (2.0)	62.0 (2.7)	C^+^	5.3	4.8	6.0	1.3
BD	15.1 (0.8)	11.9 (0.8)	20.9 (1.9)	41.2 (3.3)	N	19.2 (1.3)	17.7 (1.4)	35.8 (1.9)	53.2 (3.6)	D^+^	4.1	5.8	14.9	12.0
BW	37.1 (2.6)	36.5 (2.6)	45.7 (2.7)	40.0 (2.1)	.	40.8 (2.3)	45.0 (4.4)	61.3 (4.3)	43.1 (3.4)	C^+^	3.8	8.5	15.6	3.2
Litter														
AD	22.0 (1.0)	13.0 (1.0)	9.9 (1.2)	49.1 (2.2)	N	20.8 (1.2)	16.3 (2.2)	13.7 (1.5)	51.0 (2.7)	.	−1.3	3.2	3.8	1.9
AW	8.4 (0.9)	4.2 (0.9)	9.7 (1.9)	27.8 (2.4)	N	9.4 (1.1)	4.3 (0.8)	3.7 (0.7)	31.9 (2.5)	I	1.0	0.0	−6.0	4.1
BD	10.1 (0.7)	8.1 (0.7)	12.3 (0.9)	15.3 (1.6)	N	12.5 (1.2)	9.5 (1.5)	12.4 (1.0)	32.0 (2.6)	I	2.4	1.3	0.0	16.7
BW	27.6 (1.5)	9.4 (1.5)	26.9 (1.5)	43.2 (4.3)	N	32.8 (1.4)	13.5 (1.7)	24.7 (2)	60.2 (3.8)	I	5.2	4.1	−2.2	17.0
Open Canopy														
AD	53.0 (2.4)	61.2 (2.4)	42.8 (2.2)	45.4 (3.5)	N	54.5 (2.4)	56.3 (3.1)	46.2 (1.8)	40.5 (3.5)	.	1.5	−4.9	3.4	−5.0
AW	24.6 (1.9)	23.6 (1.9)	38.5 (1.9)	13.1 (1.7)	N	22.3 (1.9)	15.5 (1.6)	26.5 (2.3)	9.8 (1.2)	I	−2.3	−8.2	−12.0	−3.3
BD	48.4 (1.6)	35.9 (1.6)	40.0 (2.4)	30.4 (1.7)	N	41.8 (1.9)	27.9 (2.3)	24.6 (1.9)	13.6 (1.8)	D^−^	−6.6	−8.0	−15.5	−16.8
BW	15.2 (2.2)	9.6 (2.2)	28.3 (2.4)	9.8 (1.6)	N	11.3 (1.2)	8.0 (0.9)	21.3 (2.3)	6.7 (1.0)	C^−^	−3.9	−1.7	−7.0	−3.2
Species Richness														
AD	6.4 (0.2)	5.7 (0.2)	6.8 (0.2)	6.0 (0.2)	N	6.3 (0.2)	5.8 (0.2)	6.6 (0.2)	6.2 (0.2)	.	−0.1	0.1	−0.2	0.2
AW	5.0 (0.2)	4.9 (0.2)	5.3 (0.3)	5.2 (0.1)	.	4.9 (0.2)	4.8 (0.1)	5.0 (0.2)	5.1 (0.2)	.	−0.1	−0.1	−0.3	0.0
BD	5.3 (0.2)	6.5 (0.2)	6.5 (0.2)	6.0 (0.2)	N	5.5 (0.2)	6.7 (0.2)	7.5 (0.2)	6.6 (0.2)	C^+^	0.2	0.2	1.0	0.6
BW	7.3 (0.2)	7.6 (0.2)	7.3 (0.3)	6.9 (0.2)	.	7.3 (0.3)	7.4 (0.3)	7.1 (0.2)	6.3 (0.2)	.	0.0	−0.2	−0.2	−0.5
Shannon Index														
AD	0.97 (0.01)	1.02 (0.02)	0.99 (0.02)	0.92 (0.02)	D^−^	0.95 (0.01)	1.00 (0.02)	1.00 (0.01)	0.93 (0.02)	.	−0.02	−0.02	0.00	0.01
AW	0.32 (0.01)	0.37 (0.02)	0.34 (0.02)	0.35 (0.02)	N	0.33 (0.01)	0.35 (0.01)	0.31 (0.01)	0.32 (0.01)	.	0.01	−0.02	−0.02	−0.03
BD	0.76 (0.01)	0.77 (0.02)	0.80 (0.01)	0.69 (0.01)	N	0.75 (0.02)	0.71 (0.02)	0.70 (0.02)	0.63 (0.02)	D^−^	−0.01	−0.05	−0.10	−0.07
BW	0.44 (0.02)	0.44 (0.02)	0.41 (0.01)	0.37 (0.01)	N	0.41 (0.02)	0.40 (0.01)	0.40 (0.01)	0.33 (0.01)	C^−^	−0.03	−0.04	0.00	−0.04

**Table 4:** Change in canopy height over time in control plots and in response to warming at the four sites. Height was calculated as the maximum height recorded in a plot for each taxon. See Table2 for an explanation of the table layout

	C1	C2	C3	C4		E1	E2	E3	E4		W1	W2	W3	W4
Atqasuk dry (AD) site														
**Plot Maximum**	**9.6 (1.2)**	**7.0 (1.2)**	**12.7 (0.9)**	**11.0 (0.9)**	**N**	**11.7 (1.2)**	**6.4 (0.5)**	**14.9 (1.2)**	**12.5 (1.2)**	**.**	**2.1**	−**0.6**	**2.2**	**1.6**
**Evergreen Shrub**	**3.7 (0.4)**	**3.5 (0.4)**	**4.2 (0.4)**	**4.6 (0.5)**	**.**	**3.1 (0.2)**	**2.6 (0.2)**	**3.9 (0.3)**	**5.1 (0.3)**	**.**	−**0.6**	−**0.9**	−**0.3**	**0.5**
*Cassiope tetragona*	3.5 (0.4)	2.2 (0.4)	4.1 (0.4)	3.6 (0.4)	N	2.7 (0.2)	2.4 (0.2)	2.7 (0.4)	4.5 (0.3)	I	−0.8	0.1	−1.4	0.8
*Diapensia lapponica*	0.3 (0.2)	0.3 (0.2)	0.7 (0.3)	0.6 (0.3)	.	0.1 (0.0)	0.2 (0.1)	0.5 (0.2)	0.6 (0.3)	.	−0.3	−0.1	−0.2	0.0
*Ledum palustre*	2.1 (0.2)	2.3 (0.2)	1.8 (0.2)	3.2 (0.5)	N	1.9 (0.2)	1.9 (0.2)	2.9 (0.5)	4.1 (0.4)	.	−0.3	−0.4	1.1	0.9
*Vaccinium vitis-idaea*	1.1 (0.1)	1.1 (0.1)	0.5 (0.1)	1.3 (0.3)	.	1.4 (0.2)	1.0 (0.2)	0.2 (0.1)	1.2 (0.2)	.	0.2	−0.1	−0.2	0.0
**Graminoid**	**9.4 (1.3)**	**6.6 (1.3)**	**12.2 (1.0)**	**10.9 (1.0)**	**N**	**11.6 (1.3)**	**6.3 (0.5)**	**14.9 (1.2)**	**12.0 (1.2)**	**.**	**2.2**	−**0.3**	**2.7**	**1.1**
*Hierochloe alpina*	7.7 (1.6)	4.9 (1.6)	11.9 (1.3)	9.6 (1.3)	N	10.0 (1.7)	5.3 (0.6)	12.1 (1.6)	10.1 (1.4)	.	2.3	0.4	0.2	0.6
*Luzula confusa*	5.6 (0.8)	4.7 (0.8)	7.5 (0.8)	7.5 (1.0)	N	6.5 (0.9)	3.8 (0.5)	9.6 (1.2)	6.2 (0.8)	.	0.8	−0.9	2.0	−1.4
Atqasuk wet (AW) site														
**Plot Maximum**	**21.9 (1.1)**	**19.2 (1.1)**	**24.1 (1.2)**	**20.7 (1.0)**	**N**	**24.4 (0.9)**	**22.7 (0.8)**	**27.8 (1.1)**	**24.2 (1.1)**	**C**^**+**^	**2.5**	**3.5**	**3.7**	**3.5**
**Deciduous Shrub**	**9.5 (0.9)**	**10.2 (0.9)**	**10.9 (0.4)**	**11.4 (0.6)**	**.**	**10.2 (0.9)**	**12.1 (0.9)**	**13.6 (1.2)**	**12.8 (0.9)**	**C**^**+**^	**0.6**	**1.8**	**2.7**	**1.3**
*Salix pulchra*	10.0 (0.9)	9.9 (0.9)	10.6 (0.6)	11.2 (0.7)	.	10.7 (0.9)	12.2 (1.0)	13.4 (1.2)	11.9 (1.2)	C^+^	0.7	2.2	2.8	0.7
**Graminoid**	**21.9 (1.1)**	**19.2 (1.1)**	**24.1 (1.2)**	**20.5 (1.0)**	**N**	**24.4 (0.9)**	**22.7 (0.8)**	**27.7 (1.1)**	**24.2 (1.1)**	**C**^**+**^	**2.5**	**3.5**	**3.5**	**3.7**
*Carex aquatilis*	21.8 (1.2)	18.8 (1.2)	23.5 (1.3)	19.9 (1.2)	N	24.0 (0.9)	22.3 (0.8)	27.5 (1.1)	24.0 (1.1)	C^+^	2.2	3.5	4.1	4.2
*Eriophorum angustifolium*	12.0 (1.1)	12.6 (1.1)	15.7 (1.3)	12.5 (0.9)	.	14.3 (1.4)	14.6 (0.8)	16.6 (1.1)	15.3 (1.1)	C^+^	2.3	2.0	1.0	2.8
*Eriophorum russeolum*	12.6 (1.0)	9.8 (1.0)	12.3 (1.1)	10.7 (1.1)	.	13.2 (1.0)	14.0 (1.1)	14.4 (1.1)	13.0 (1.0)	C^+^	0.6	4.2	2.2	2.3
Barrow Dry (BD) site														
**Plot Maximum**	**3.6 (0.4)**	**6.0 (0.4)**	**8.1 (0.7)**	**7.9 (0.6)**	**D**^**+**^	**6.6 (0.6)**	**9.3 (1.4)**	**12.8 (0.7)**	**12.7 (0.8)**	**D**^**+**^	**2.9**	**3.3**	**4.6**	**4.8**
**Deciduous Shrub**	**0.1 (0.1)**	**1.3 (0.1)**	**0.6 (0.2)**	**1.8 (0.1)**	**N**	**0.3 (0.1)**	**1.6 (0.2)**	**0.2 (0.1)**	**2.2 (0.2)**	**.**	**0.2**	**0.3**	−**0.4**	**0.4**
*Salix rotundifolia*	0.1 (0.1)	1.3 (0.1)	0.6 (0.2)	1.8 (0.1)	N	0.3 (0.1)	1.6 (0.2)	0.2 (0.1)	2.2 (0.2)	.	0.2	0.3	−0.4	0.4
**Evergreen Shrub**	**2.1 (0.5)**	**3.1 (0.5)**	**4.2 (0.2)**	**5.4 (0.3)**	**D**^**+**^	**3.3 (0.5)**	**4.1 (0.3)**	**5.2 (0.3)**	**7.9 (0.5)**	**D**^**+**^	**1.2**	**1.0**	**0.9**	**2.5**
*Cassiope tetragona*	2.1 (0.5)	3.1 (0.5)	4.2 (0.2)	5.4 (0.3)	D^+^	3.3 (0.5)	4.1 (0.3)	5.2 (0.3)	7.9 (0.5)	D^+^	1.2	1.0	0.9	2.5
**Forb**	**1.1 (0.4)**	**3.0 (0.4)**	**4.9 (0.8)**	**5.1 (0.8)**	**D**^**+**^	**3.5 (0.7)**	**4.4 (1.2)**	**7.0 (1.2)**	**7.8 (1.2)**	**D**^**+**^	**2.4**	**1.4**	**2.1**	**2.8**
*Potentilla hyparctica*	1.0 (0.6)	2.5 (0.6)	3.3 (0.8)	3.2 (0.5)	D^+^	2.2 (0.5)	1.0 (0.5)	6.9 (1.2)	8.7 (1.2)	D^+^	1.2	−1.5	3.6	5.5
**Graminoid**	**2.1 (0.4)**	**4.9 (0.4)**	**6.6 (0.6)**	**5.9 (0.5)**	**N**	**4.0 (0.7)**	**7.0 (1.3)**	**11.7 (0.7)**	**10.5 (0.8)**	**D**^**+**^	**1.9**	**2.1**	**5.1**	**4.7**
*Luzula confusa*	1.3 (0.4)	3.2 (0.4)	4.9 (0.4)	4.2 (0.4)	D^+^	2.2 (0.4)	2.8 (0.4)	7.2 (0.8)	6.6 (0.5)	D^+^	0.8	−0.4	2.3	2.4
*Poa arctica*	0.2 (0.2)	2.4 (0.2)	4.2 (0.6)	3.9 (0.5)	D^+^	0.6 (0.2)	3.2 (0.5)	8.9 (0.9)	8.5 (0.9)	D^+^	0.4	0.8	4.7	4.6
Barrow Wet (BW) site														
**Plot Maximum**	**8.9 (0.6)**	**11.4 (0.6)**	**13.1 (0.6)**	**14.2 (0.4)**	**D**^**+**^	**11.4 (0.5)**	**12.9 (0.6)**	**15.0 (0.5)**	**16.8 (0.8)**	**D**^**+**^	**2.5**	**1.6**	**1.9**	**2.7**
**Forb**	**3.5 (0.5)**	**3.4 (0.5)**	**5.4 (0.9)**	**7.0 (0.9)**	**D**^**+**^	**5.6 (0.9)**	**3.2 (0.6)**	**7.4 (1.2)**	**7.8 (1.1)**	**.**	**2.1**	−**0.1**	**2.0**	**0.8**
*Saxifraga cernua*	0.4 (0.2)	0.9 (0.2)	2.7 (1.0)	3.8 (1.3)	D^+^	1.5 (0.6)	0.8 (0.4)	2.5 (1.2)	4.4 (1.0)	.	1.1	−0.1	−0.2	0.6
*Stellaria laeta*	2.1 (0.6)	2.3 (0.6)	3.0 (0.4)	2.8 (0.5)	.	3.7 (0.6)	2.1 (0.7)	3.0 (0.6)	3.8 (0.6)	.	1.6	−0.2	0.0	1.0
**Graminoid**	**8.9 (0.6)**	**11.4 (0.6)**	**12.8 (0.5)**	**14.1 (0.4)**	**D**^**+**^	**10.5 (0.6)**	**12.9 (0.6)**	**14.7 (0.5)**	**16.4 (0.8)**	**D**^**+**^	**1.6**	**1.6**	**1.9**	**2.3**
*Carex aquatilis*	8.0 (0.5)	9.9 (0.5)	11.0 (0.6)	12.7 (0.4)	D^+^	10.3 (0.7)	12.7 (0.6)	14.0 (0.4)	14.8 (0.6)	D^+^	2.3	2.8	3.0	2.1
*Dupontia fisheri*	6.6 (0.7)	9.0 (0.7)	10.6 (0.7)	11.3 (0.6)	D^+^	6.1 (0.6)	8.1 (0.7)	10.5 (0.8)	12.5 (1.0)	.	−0.5	−0.9	−0.1	1.1
*Eriophorum angustifolium*	3.9 (0.4)	7.6 (0.4)	6.6 (0.7)	9.4 (0.4)	N	4.9 (0.5)	8.6 (0.8)	9.0 (0.9)	9.9 (0.7)	C^+^	1.1	1.0	2.4	0.5
*Eriophorum russeolum*	2.6 (0.3)	4.4 (0.3)	6.7 (0.4)	6.0 (0.5)	D^+^	2.4 (0.5)	5.3 (0.8)	8.5 (0.7)	7.6 (0.8)	D^+^	−0.2	0.9	1.8	1.6
Poaceae spp.^1^	4.2 (0.5)	4.7 (0.5)	6.8 (0.7)	8.4 (1.3)	D^+^	3.6 (0.5)	4.3 (0.5)	8.4 (0.9)	10.0 (1.3)	.	−0.5	−0.4	1.6	1.5

1 *Calamagrostis holmii, Hierochloe pauciflora, Poa arctica*.

At the AW site, the canopy height of all the shrubs and graminoids consistently increased with warming, but these differences were realized at the first sampling and remained relatively constant (Table4). Changes in the cover of graminoids, bryophytes, total live plants, litter, and open canopy were nondirectional over time and inconsistent with warming (Tables2 and 3, Fig.4). Changes in the cover of standing dead and the canopy height of graminoids were nondirectional over time. Cover of standing dead increased with warming.

At the BD site, canopy height increased over time and with warming (Tables3 and 4, Fig.4). Canopy height of evergreen shrubs, forbs, and the dominant graminoid species increased over time and with warming, resulting in more than a doubling of maximum canopy height over the 18 years of sampling (Table4). *Poa arctica* was particularly responsive and increased both canopy height and cover over time and with warming (Tables2 and 4). Changes in the cover of graminoids, total live plants, and litter were nondirectional over time and inconsistent with warming. Changes in the cover of shrubs, forbs, bryophytes, lichens, and open canopy and species richness were nondirectional over time, while the cover of deciduous shrubs, bryophytes, lichens, and open canopy and diversity decreased and the cover of evergreen shrubs and standing dead and species richness increased with warming.

At the BW site, the canopy height of graminoids increased over time and with warming (Tables3 and 4, Fig.4). Cover of total live plants and litter was nondirectional over time and inconsistent with warming (Table3, Fig.4). Forbs decreased over time, and changes in the cover of graminoids, bryophytes, and open canopy were nondirectional over time (Tables2 and 3, Fig.4). Cover of deciduous shrubs and standing dead increased, while cover of bryophytes, lichens, and open canopy and diversity decreased with warming. Cover of graminoids and forbs did not change significantly with warming despite significant changes in species within each group. Height of forbs increased over time but did not change with warming.

### Comparisons across sites

The number of taxa that showed significant changes in cover over time was greater in the control plots than in response to warming (39 taxa vs. 28 of 58, Tables2 and 5). However, in the control plots, only five taxa showed a directional change; the rest (34) changed in ways that were inconsistent across years and therefore nondirectional. Of these, the four that decreased were in either the AD site or BW site, and they included one forb and three lichens; these taxa also decreased with warming except the forb at the BW site. The only taxon that increased in the control plots was a grass, *Poa arctica*, at the BD site, which also increased with warming. With warming, fewer taxa changed, but of the ones that did, all but five showed either a cumulative directional change (13 taxa) or a consistent change (10 taxa). The AW site was the only site where no taxa showed a consistent warming response. At the AD site, one taxon increased and three decreased; at the BD site, three taxa increased and seven decreased; and at the BW site, three taxa increased and six decreased. Taxa that increased with warming included one deciduous shrub, three evergreen shrubs, one forb, and two graminoids. Taxa that decreased with warming included two deciduous shrubs, one graminoid, five bryophytes, and eight lichens. Of the taxa that showed a cumulative directional change with warming, shrubs and graminoids increased and bryophytes and lichens decreased.

**Table 5:** Summary of the consistency of changes over time in cover of taxa from Table2. The changes in control plots (ambient) between years and in response to experimental warming (warmed) are shown. The table tabulates the number of taxa categorized as no change (.), changed nondirectionally (N), changed inconsistently (I), changed consistently (decrease C^−^ or increase C^+^), and changed directionally over time (decrease D^−^ or increase D^+^) grouped by site and growth form

	Ambient				Warmed					
	.	N	D^−^	D^+^	.	I	C^−^	C^+^	D^−^	D^+^
Site										
Atqasuk Dry	5	8	3	0	12	0	1	0	2	1
Atqasuk Wet	8	4	0	0	9	3	0	0	0	0
Barrow Dry	2	12	0	1	3	2	4	0	3	3
Barrow Wet	4	10	1	0	6	0	2	3	4	0
Growth Form										
Deciduous Shrub	4	2	0	0	3	0	2	1	0	0
Evergreen Shrub	2	5	0	0	4	0	0	0	0	3
Forb	4	2	1	0	6	0	0	1	0	0
Graminoid	3	12	0	1	10	3	1	1	0	1
Bryophyte	2	10	0	0	5	2	3	0	2	0
Lichen	4	3	3	0	2	0	1	0	7	0
Total	19	34	4	1	30	5	7	3	9	4

The community indices generally showed nondirectional changes over time except species diversity which decreased at the AD site (Table3). With warming, there was an increase in the cover of standing dead at all sites and a decrease in the cover of open canopy and diversity at the two sites in Barrow and an increase in the number of vascular taxa at the BD site.

Changes in canopy height were greater at Barrow than Atqasuk (Table4). In Atqasuk, there was either no change or an inconsistent change in height over time and with warming, except at the wet site which showed an increase in canopy height for all taxa with warming only, however, this change was observed in the first sampling and was not cumulative. At Barrow, most, but not all, taxa showed a cumulative increase in canopy height over time and with warming.

In summary, from an examination of the tables, it is clear that the magnitude of change was almost always greater between years than in response to experimental warming. In addition, the number of significant responses was greater over time than with warming. However, the change over time was mostly nondirectional. Of the 21 instances where change over time was directional (this included changes in community indices and cover of taxa), all but six showed a corresponding directional change with warming.

## Discussion

Temperature trends in Barrow and Atqasuk regions followed similar trends to those found elsewhere in high latitude regions (Serreze et al. 2000; ACIA 2005; IPCC 2013). Both regions had variability in mean July temperatures between years with a small increasing trend across the duration of the study. While this trend was not statistically significant, it was consistent with documented trends (earlier snowmelt and warmer summers) in the region (Stone et al. 2002; Hinzman et al. 2005; Lynch and Brunner 2007; Wendler et al. 2014).

Overall, the vegetative changes in control plots between samplings were larger than the responses to warming. This should be expected given that temperature varied more between years than in response to experimental warming, and precipitation and soil moisture varied greatly between years as did herbivore intensity (the impact of herbivory was documented in adjacent areas by Villarreal et al. (2012) and shown to be a strong determinant of plant community composition). In most cases, changes in control plots were inconsistent through time, whereas responses to warming, while fewer, were mostly consistent. A directional change is likely due to a clear competitive advantage (or disadvantage) resulting in a cumulative increase (or decrease) over time. A consistent response may be due to a release from temperature restraints that causes a physiological response which does not accumulate, such as an increase in growth/biomass of a preexisting individual.

The instances where directional changes in the control plots were matched by a directional change in response to warming provide strong evidence that the observed change in the control plots is due to warming. These included a decrease in cover of lichens at the AD site, increased canopy height at both sites in Barrow, and an increase in canopy height and cover of *Poa arctica* at the BD site. A decrease in the abundance of lichens is consistent with the majority of warming studies (Cornelissen et al. 2001; Elmendorf et al. 2012a; Lang et al. 2012). Canopy height has been shown by a number of studies to increase with warming and across natural temperature gradients (Walker et al. 2006; Elmendorf et al. 2012a), and in fact is one of the most consistent responses seen when examining warming studies (Elmendorf et al. 2012a). *Poa arctica* is commonly associated with disturbance (Potter 1972; Bliss and Peterson 1992) and may be responding directly to warming or indirectly to disturbances caused by warming which may include increased nutrient availability given that the species is generally more common in fertilized areas (Gartner et al. 1983).

The warming experiment provides a possible look into future vegetation at the sites. From this, we would expect over the next two decades to see further changes in the control plots in addition to those already observed assuming the region continues to warm. These include decreases in the cover of lichens at all sites except the AW site (which is already nearly devoid of lichens), decreases in bryophytes at the BW site, and an increase in the cover of evergreen shrub, *Cassiope tetragona* at the dry sites, an increase in standing dead at the BD site, a decrease in the cover of open canopy at the BD site, and a decrease in diversity at the BD site. Decreases in bryophytes with warming, while originally proposed, have been recently questioned; mechanistically an increase in stature of vascular plants may benefit mosses by providing shade (Zona et al. 2011; Jägerbrand et al. 2012). Thus, the mixed result shown here of some sites showing a decrease in the cover of moss and others showing no response is consistent with recent studies (Lang et al. 2012). The increased cover of *Cassiope tetragona* with warming is consistent with a large volume of literature that has examined the species (Havström et al. 1993; Weijers et al. 2012); in fact, the species is often used as a climate proxy because of its tight coupling between growth and seasonal temperature (Callaghan et al. 1989; Weijers et al. 2012). The increased cover of standing dead with warming is consistent with previously accepted ideas of arctic plants holding their dead leaves in the canopy (Bliss 1962; Savile 1972) and is consistent with warming experiments that have found similar increases (Elmendorf et al. 2012a). Increased standing dead may be due to increased growth in the early years of the experiment and the resulting growth senescing then being retained as standing dead. The decrease in the openness of the canopy has been less well documented, but given that the consensus findings are increases in graminoids and shrubs and decreases in lichens with warming (Elmendorf et al. 2012a), this is consistent with a general loss of open space in the canopy. It is important to note that the Barrow sites, where the canopy became less open with warming, have very short canopy heights, and the open canopy is a more or less colonizable area for vascular plants, whereas at Atqasuk, the canopy height is much taller and the open area is heavily shaded (Fig.1). Diversity of vascular plants is expected to ultimately increase in tundra with warming (Walker 1995; Francis and Currie 2003); however, this study and a synthesis of experimental warming studies in tundra have shown a decrease in diversity (Walker et al. 2006) or no change (Elmendorf et al. 2012a).

Predicting community change based on growth form may be problematic. At several sites, the taxa within growth forms increased while others decreased resulting in a muted warming response; this was especially true at the BW site. This disparity in how taxa within growth forms respond may be explained by grouping taxa by other attributes, such as home range, maximum plant height, or leaf density (Cornelissen et al. 2003; Kattge et al. 2011). Such grouping schemes, or a suite of them, may better identify traits that respond similarly and make predicting community changes in response to changing environmental conditions more accurate (Suding et al. 2008; Dorji et al. 2013; Soudzilovskaia et al. 2013).

Variability in weather, especially temperature, between years may explain much of the nondirectional change observed over time (Chapin and Shaver 1996; Arft et al. 1999). Microclimate differences within sites could also allow for conditions between plots to vary enough that a species may be successful in some plots and not others (Hudson and Henry 2009). Confounding effects may have led to variations in warming responses between years (Walker et al. 1994; Cooper et al. 2011). For example, it appears that experimental warming is in general limiting growth at the AD site and it is likely this is because the site is water stressed (especially in later years) and temperature is not as limiting a factor, whereas at the other three sites, canopy height is clearly responsive to warming. Nontemperature factors may prove helpful in the future when incorporated into investigations about arctic plant community changes (Phoenix and Lee 2004). However, it is difficult to separate the drivers of directional change from the many factors that fluctuate between years without long-term repeated annual sampling. Furthermore, the cumulative nature of directional change makes it difficult to correlate change in community composition to factors other than year.

This study shows the power of coupling an in situ experiment with long-term monitoring. Clearly in most cases, species fluctuate between years in ways that are difficult to decipher. However, in cases where there are clear directional changes in natural communities, it is not possible to identify the driver without additional information. Therefore, when it is important to identify the driving factors at a given site, we advocate for coupling long-term monitoring with in situ experiments. This is especially true in cases where results from an intensely studied site are generalized to a much larger region. Assuming that manipulation is low cost and logistically simple, the addition of manipulations can add greatly to the utility of new and existing monitoring programs. Monitoring programs such as these are needed to inform policy decisions as ecologists grapple with global change.

## Data Accessibility

All data are available at ACADIS managed by the National Snow and Iced Data Center (http://nsidc.org/acadis/).
